# Supplementary material for: Propagation Capacity of Phage Display Peptide Libraries Is Affected by the Length and Conformation of Displayed Peptide
Source: Molecules. 2023 Jul 10;28(14):5318. doi: 10.3390/molecules28145318 (PMC10386350; doi:10.3390/molecules28145318)
Supplement: Supplementary file 1 [file molecules-28-05318-s001.zip › molecules-2458644-supplementary.pdf]

Supplementary material

Table S1. ***Number of NGS reads at different time points obtained from competitive propagation assay*** *The table indicates the total number of reads obtained after Illumina sequencing, and the number of cleaned and removed reads after applying MATLAB and Python scripts to the raw data of high-throughput sequencing.*

Time point (min)	Total Number of Reads	Number of Removed Reads	Number of Cleaned Reads	Percentage of Removed Reads
0	168,648	35,000	133,648	20.8
150	288,715	45,016	243,699	15.6
270	585,628	94,510	491,118	16.1

Table S2. ***Top 30 sequences sorted based on enrichment factor obtained from competitive propagation assay*** *The table reveals that although Ph.D.-7 library had the highest propagation capacity on a population level, some phage clones from the two other libraries also showed high enrichment factors.*

Sequence	Enrichment factor	Library
GNMGYMRPGHNN	82,87	Ph.D.-12
QWTVVTP	49,13	Ph.D.-7
VPLIQSH	47,14	Ph.D.-7
ACGQQKGNSC	38,70	Ph.D.-C7C
ACSKGSPGDC	37,22	Ph.D.-C7C
ACTDKASSSC	37,22	Ph.D.-C7C
HATGYTKPNVHQ	36,22	Ph.D.-12
ACTPRSANYC	33,25	Ph.D.-C7C
APVRTHF	29,28	Ph.D.-7
SEQVLPPSWKTT	28,78	Ph.D.-12
ACKGPMAVMC	28,78	Ph.D.-C7C
GPREGATSCCSV	28,78	Ph.D.-12
WPTVSQT	27,29	Ph.D.-7
SPQAPTNSMYWD	26,80	Ph.D.-12
TGAAEVAMMSKR	25,68	Ph.D.-12
QSYFNACWSCNH	24,78	Ph.D.-12
SVYNALYLAASE	24,49	Ph.D.-12
EQPGVVDPFVGR	23,32	Ph.D.-12
LPHQFHVASNDN	23,32	Ph.D.-12
TFNNFYEARTEL	22,83	Ph.D.-12
YSSLGNS	22,33	Ph.D.-7
AAISPSH	22,33	Ph.D.-7
TDYWSLK	22,33	Ph.D.-7
TAWASAA	21,83	Ph.D.-7
SPYHDPHYTDKF	21,83	Ph.D.-12
ACDHEKPGRC	21,34	Ph.D.-C7C

ACKGTSMRTC	21,34	Ph.D.-C7C
ATNYLVT	21,34	Ph.D.-7
ASQASAH	21,34	Ph.D.-7

Table S3. ***MATLAB® script (Load_NGS_Data_No_X_no_indeces.m).*** *The MATLAB script translated the NGS data (FASTQ files) from nucleotides into amino acid (AA) sequences in the region of interest (the peptide insert) and with the size of interest (16 AAs). These peptide sequence reads were then filtered into either cleaned or removed reads based on multiple variables such as the presence of the linker sequence GGGS, location of the linker sequence, presence of untranslatable AA's, etc. The script was run in MATLAB R2022a.*

```
function [AA_Clean, AA_Removed, Reads_Clean, Reads_Removed, Reads_RC] = ...
  Load_NGS_Data_No_X_no_indeces(FileName, Library, WriteToExcel)

  %%% Library chosen decides length of sequences to load %%%
  if strcmp(Library, 'PhD7')
    Seq_Length = 7 + 4;
    Seq_Nucleotides = [1:33];
  elseif strcmp(Library, 'PhD12')
    Seq_Length = 12 + 4;
    Seq_Nucleotides = [1:48];
  elseif strcmp(Library, 'PhDC7C')
    Seq_Length = 3 + 7 + 4;
    Seq_Nucleotides = [1:42];
  else
    error('Use either PhD7, PhD12, or PhDC7C.');
  end

  %%% Read the sequences from the fastq file %%%
  disp('Reading in fastq file');
  [~, Seqs, ~] = fastqread(FileName);

  %%% Removing flanking region
  disp('Removing flanking regions');
  Seqs_Nucleotide = cell(length(Seqs), 1);
  Seqs_Error_Length = cell(length(Seqs), 1);

  for i = 1 : length(Seqs)

    if length(Seqs{i}) >= Seq_Nucleotides(end)
      Seqs_Nucleotide{i} = Seqs{i}(Seq_Nucleotides);
    else
      Seqs_Error_Length{i} = Seqs{i};
    end

  end

  Seqs_Nucleotide = Seqs_Nucleotide(~cellfun('isempty', Seqs_Nucleotide));
  Seqs_Error_Length = Seqs_Error_Length(~cellfun('isempty', Seqs_Error_Length));
```

```
%%% Convert nucleotides to AA and put into a frequency sorted matrix %%%
disp('Conversion to amino acids');
AA_Array = nt2aa(Seqs_Nucleotide,...
   'AlternativeStartCodons',false,...
   'ACGTonly', false);

disp('Tabulating amino acids');
AA_Cell = tabulate(AA_Array);


%%%% Clean up data by ensuring proper end and lack of '*' %%%%%
disp('Cleaning up the sequences.');

%Remove invalid '*' sequences
disp('Removing sequences with *.');
InvIdx1 = strfind(AA_Cell(:, 1), '*');

AA_Removed = AA_Cell(~cellfun('isempty',InvIdx1),:);
AA_Clean = AA_Cell(cellfun('isempty',InvIdx1),:);

%Ensure proper ending, i.e., 'GGGS'
disp('Ensuring proper ending of GGGS.');
InvIdx2 = strfind(AA_Clean(:, 1), 'GGGS');
InvIdx2 = cellfun(@(InvIdx2) find(InvIdx2 == Seq_Length - 3),...
   InvIdx2, 'UniformOutput', false);

AA_Removed = [AA_Removed; AA_Clean(cellfun('isempty', InvIdx2), :)];
AA_Clean = AA_Clean(~cellfun('isempty', InvIdx2), :);

%Remove invalid 'X' sequences
disp('Removing sequences with X.');
InvIdx3 = strfind(AA_Clean(:, 1), 'X');

AA_Removed = [AA_Removed; AA_Clean(~cellfun('isempty', InvIdx3), :)];
AA_Clean = AA_Clean(cellfun('isempty', InvIdx3), :);


if strcmp(Library, 'PhDC7C')

   %Need to check for 'AC' beginning
   InvIdx4 = strfind(AA_Clean(:, 1), 'AC');
   InvIdx4 = cellfun(@(InvIdx4) find(InvIdx4 == 1),...
      InvIdx4, 'UniformOutput', false);


   AA_Removed = [AA_Removed; AA_Clean(cellfun('isempty', InvIdx4), :)];
   AA_Clean = AA_Clean(~cellfun('isempty', InvIdx4), :);

   %and 'C' after sequence
   InvIdx5 = strfind(AA_Clean(:, 1), 'C');
   InvIdx5 = cellfun(@(InvIdx5) find(InvIdx5 == 10),...
```

```
        InvIdx5, 'UniformOutput', false);

      AA_Removed = [AA_Removed; AA_Clean(cellfun('isempty', InvIdx5), :)];
      AA_Clean = AA_Clean(~cellfun('isempty', InvIdx5), :);

   end


   %Sort the cells
   AA_Clean = sortrows(AA_Clean, -2);
   AA_Removed = sortrows(AA_Removed, -2);

   %Update relative percentage for clean and removed lists
   AA_Clean(:, 3) = num2cell(100 * cell2mat(AA_Clean(:, 2)) /...
      sum(cell2mat(AA_Clean(:, 2))));
   AA_Removed(:, 3) = num2cell(100 * cell2mat(AA_Removed(:, 2)) /...
      sum(cell2mat(AA_Removed(:, 2))));

   %%% Calculate the number of reads %%%
   Reads_Clean = sum(cell2mat(AA_Clean(:,2)));
   Reads_Removed = sum(cell2mat(AA_Removed(:,2)));
   Reads_RC = Reads_Removed / (Reads_Clean + Reads_Removed);

   %%% Decide upon writing to an excel file %%%
   if ~strcmp(WriteToExcel, 'No')
      %Limit set to 100,000 sequences - can be increased to max 8,640,909
      Seq_Limit = 1:10^3;

%        AA_Clean_Table = table(AA_Clean(Seq_Limit,1),...
%                          cell2mat(AA_Clean(Seq_Limit,2)),...
%                          cell2mat(AA_Clean(Seq_Limit,3)),...
%                      'VariableNames', {'AA', 'Number', 'Relative (%)'});
%        AA_Removed_Table = table(AA_Removed(Seq_Limit,1),...
%                          cell2mat(AA_Removed(Seq_Limit,2)),...
%                          cell2mat(AA_Removed(Seq_Limit,3)),...
%                      'VariableNames', {'AA', 'Number', 'Relative (%)'});
%
%
%        writetable(AA_Clean_Table, WriteToExcel + "_AA_Clean.xls");
%        writetable(AA_Removed_Table, WriteToExcel + "_AA_Removed.xls");

      AA_Clean_Table = table(AA_Clean(:,1),...
                        cell2mat(AA_Clean(:,2)),...
                        cell2mat(AA_Clean(:,3)),...
                    'VariableNames', {'AA', 'Number', 'Relative (%)'});
      AA_Removed_Table = table(AA_Removed(:,1),...
                        cell2mat(AA_Removed(:,2)),...
                        cell2mat(AA_Removed(:,3)),...
                    'VariableNames', {'AA', 'Number', 'Relative (%)'});
```

```
      writetable(AA_Clean_Table, WriteToExcel + "_AA_Clean.csv");
      writetable(AA_Removed_Table, WriteToExcel + "_AA_Removed.csv");
   end

end
```

Table S4. ***Python script 1 (Removal of duplicates_removed.py).*** *The table contains the python script used to identify and remove duplicate sequences between the MATLAB script-generated removed reads of the three different libraries and the pooling of the non-duplicate reads into non-library specific removed reads for each timepoint. The script was developed and run in Spyder (Python 3.9).*

```
# -*- coding: utf-8 -*-
"""
Created on Thur Dec 01 12:28:51 2022

@author: zmn159
"""


import os
import pandas as pd

''' Script to remove duplicates common between the removed and clean sequences (necessary for all libs)'''

# Desktop location:
data_root = r'C:\Users\zmn159\OneDrive - University of Copenhagen\Desktop\DKS_size_comp\csv files (new)'

''' Paths - Only have a group of three active at a time!'''

one_root = os.path.join(data_root, 'DKS_006_2109_t0_R1_Phd7_AA_Removed.csv')
two_root = os.path.join(data_root, 'DKS_006_2109_t0_R1_PhdC7C_AA_Clean.csv')
three_root = os.path.join(data_root, 'DKS_006_2109_t0_R1_Phd12_AA_Clean.csv')

# one_root = os.path.join(data_root, 'DKS_006_2109_t0_R1_Phd7_AA_Clean.csv')
# two_root = os.path.join(data_root, 'DKS_006_2109_t0_R1_PhdC7C_AA_Removed.csv')
# three_root = os.path.join(data_root, 'DKS_006_2109_t0_R1_Phd12_AA_Clean.csv')

# one_root = os.path.join(data_root, 'DKS_006_2109_t0_R1_Phd7_AA_Clean.csv')
# two_root = os.path.join(data_root, 'DKS_006_2109_t0_R1_PhdC7C_AA_Clean.csv')
# three_root = os.path.join(data_root, 'DKS_006_2109_t0_R1_Phd12_AA_Removed.csv')

# one_root = os.path.join(data_root, 'DKS_006_2109_t150_R1_Phd7_AA_Removed.csv')
# two_root = os.path.join(data_root, 'DKS_006_2109_t150_R1_PhdC7C_AA_Clean.csv')
# three_root = os.path.join(data_root, 'DKS_006_2109_t150_R1_Phd12_AA_Clean.csv')

# one_root = os.path.join(data_root, 'DKS_006_2109_t150_R1_Phd7_AA_Clean.csv')
# two_root = os.path.join(data_root, 'DKS_006_2109_t150_R1_PhdC7C_AA_Removed.csv')
# three_root = os.path.join(data_root, 'DKS_006_2109_t150_R1_Phd12_AA_Clean.csv')
```

```
# one_root = os.path.join(data_root, 'DKS_006_2109_t150_R1_Phd7_AA_Clean.csv')
# two_root = os.path.join(data_root, 'DKS_006_2109_t150_R1_PhdC7C_AA_Clean.csv')
# three_root = os.path.join(data_root, 'DKS_006_2109_t150_R1_Phd12_AA_Removed.csv')

# one_root = os.path.join(data_root, 'DKS_006_2109_t270_R1_Phd7_AA_Removed.csv')
# two_root = os.path.join(data_root, 'DKS_006_2109_t270_R1_PhdC7C_AA_Clean.csv')
# three_root = os.path.join(data_root, 'DKS_006_2109_t270_R1_Phd12_AA_Clean.csv')

# one_root = os.path.join(data_root, 'DKS_006_2109_t270_R1_Phd7_AA_Clean.csv')
# two_root = os.path.join(data_root, 'DKS_006_2109_t270_R1_PhdC7C_AA_Removed.csv')
# three_root = os.path.join(data_root, 'DKS_006_2109_t270_R1_Phd12_AA_Clean.csv')

# one_root = os.path.join(data_root, 'DKS_006_2109_t270_R1_Phd7_AA_Clean.csv')
# two_root = os.path.join(data_root, 'DKS_006_2109_t270_R1_PhdC7C_AA_Clean.csv')
# three_root = os.path.join(data_root, 'DKS_006_2109_t270_R1_Phd12_AA_Removed.csv')

'''Load data'''
row_num = 10000000

one_df = pd.read_csv(one_root, nrows=row_num)
two_df = pd.read_csv(two_root, nrows=row_num)
three_df = pd.read_csv(three_root, nrows=row_num)

'''Concats the df's into one new df where all duplicated AA's are deleted from'''
concatted_df = pd.concat([one_df, two_df, three_df], axis=0)
dropduplicates_df = concatted_df['AA'].drop_duplicates(keep=False)
# duplicates_df = concatted_df.AA[concatted_df.AA.duplicated(keep=False)]

'''The remaining AA's are merged on the library related removed file to create the true removed
file'''
# Remember to change the df you merge on (one_df = PhD7, two_df = PhDC7C and three_df =
PhD12)
removed_df = pd.merge(dropduplicates_df, one_df, on="AA")

removed_df.to_csv('DKS_006_2109_t0_R1_AA_Removed_py.csv')
```

Table S5. *Python script 2 (Removal of duplicates_Clean PhD7.py).* *The table contains the python script used to identify and remove duplicate reads between the MATLAB script-generated library-specific cleaned reads of Ph.D.-7 and -12 and the creation of new Ph.D.-7 library-specific reads without duplicate Ph.D.-12 reads for each timepoint. The script was developed and run in Spyder (Python 3.9).*

```
# -*- coding: utf-8 -*-
"""
Created on Thur Dec 01 12:28:51 2022

@author: zmn159
"""


import os
import pandas as pd
```

```
''' Script to remove duplicates common between clean libraries (only a problem for PhD7)'''

# Desktop location:
data_root = r'C:\Users\zmn159\OneDrive - University of
Copenhagen\Desktop\DKS_size_comp\csv files (new)'

''' Paths - Only have a group of three active at a time!'''

# one_root = os.path.join(data_root, 'DKS_006_2109_t0_R1_Phd7_AA_Clean.csv')
# two_root = os.path.join(data_root, 'DKS_006_2109_t0_R1_PhdC7C_AA_Clean.csv')
# three_root = os.path.join(data_root, 'DKS_006_2109_t0_R1_Phd12_AA_Clean.csv')

one_root = os.path.join(data_root, 'DKS_006_2109_t150_R1_Phd7_AA_Clean.csv')
two_root = os.path.join(data_root, 'DKS_006_2109_t150_R1_PhdC7C_AA_Clean.csv')
three_root = os.path.join(data_root, 'DKS_006_2109_t150_R1_Phd12_AA_Clean.csv')

# one_root = os.path.join(data_root, 'DKS_006_2109_t270_R1_Phd7_AA_Clean.csv')
# two_root = os.path.join(data_root, 'DKS_006_2109_t270_R1_PhdC7C_AA_Clean.csv')
# three_root = os.path.join(data_root, 'DKS_006_2109_t270_R1_Phd12_AA_Clean.csv')

'''Load data'''
row_num = 10000000

# files
one_df = pd.read_csv(one_root, nrows=row_num)
two_df = pd.read_csv(two_root, nrows=row_num)
three_df = pd.read_csv(three_root, nrows=row_num)

'''Concats the df's into one new df where all duplicated AA's are deleted from'''
concatted_df = pd.concat([one_df, two_df, three_df], axis=0)
dropduplicates_df = concatted_df['AA'].drop_duplicates(keep=False)

'''The remaining AA's are merged on the library related clean file to create the true clean file of
PhD7 without duplicates present'''
# Remember to change the df you merge on (one_df = PhD7, two_df = PhDC7C and three_df =
PhD12)
removed_df = pd.merge(dropduplicates_df, one_df, on="AA")

removed_df.to_csv('DKS_006_2109_t150_R1_PhD7_AA_Clean_py.csv')
```

Table S6. ***Python script 3 (Find_WT.py).*** *The table contains the python script used to identify the number of reads related to insertless clones not displaying a peptide (WT). The script located all reads containing the sequence AETVESCLAKSH and its close variants (differing by one amino acid). The script was used on all of the time point-specific removed reads generated from python script 1 (see Table S4). The script was developed and run in Spyder (Python 3.9).*

```
# -*- coding: utf-8 -*-
"""
```

```
Created on Mon Dec 19 12:46:16 2022

@author: zmn159
"""

import os
import pandas as pd

'''Find all WT + WT one AA difference misreads'''

# Desktop location:
data_root = r'C:\Users\zmn159\OneDrive - University of Copenhagen\Desktop\DKS_size_comp\Python csv files (new)'

# data_root = r'C:\Users\Anders\OneDrive\Skrivebord\DKS_size_comp\Python csv files (new)'

'''Paths - the removed files for each library at equal time points are identical'''
t0_root = os.path.join(data_root, 'DKS_006_2109_t0_R1_Phd12_AA_Removed_py.csv')

t150_root = os.path.join(data_root, 'DKS_006_2109_t150_R1_Phd12_AA_Removed_py.csv')

t270_root = os.path.join(data_root, 'DKS_006_2109_t270_R1_Phd12_AA_Removed_py.csv')

'''Load data'''
row_num = 1000000

# only have one section at a time active!

one_df = pd.read_csv(t0_root, nrows=row_num)
two_df = pd.read_csv(t0_root, nrows=row_num)
three_df = pd.read_csv(t0_root, nrows=row_num)
four_df = pd.read_csv(t0_root, nrows=row_num)
five_df = pd.read_csv(t0_root, nrows=row_num)
six_df = pd.read_csv(t0_root, nrows=row_num)
seven_df = pd.read_csv(t0_root, nrows=row_num)
OG_df = pd.read_csv(t0_root, nrows=row_num)
N=41660+65170+26818

# one_df = pd.read_csv(t150_root, nrows=row_num)
# two_df = pd.read_csv(t150_root, nrows=row_num)
# three_df = pd.read_csv(t150_root, nrows=row_num)
# four_df = pd.read_csv(t150_root, nrows=row_num)
# five_df = pd.read_csv(t150_root, nrows=row_num)
# six_df = pd.read_csv(t150_root, nrows=row_num)
# seven_df = pd.read_csv(t150_root, nrows=row_num)
# OG_df = pd.read_csv(t150_root, nrows=row_num)
# N=84747+111135+47817

# one_df = pd.read_csv(t270_root, nrows=row_num)
# two_df = pd.read_csv(t270_root, nrows=row_num)
# three_df = pd.read_csv(t270_root, nrows=row_num)
```

```
# four_df = pd.read_csv(t270_root, nrows=row_num)
# five_df = pd.read_csv(t270_root, nrows=row_num)
# six_df = pd.read_csv(t270_root, nrows=row_num)
# seven_df = pd.read_csv(t270_root, nrows=row_num)
# OG_df = pd.read_csv(t270_root, nrows=row_num)
# N=200431+213051+77636

'''Delete redundant columns from df's'''
one_df = one_df.drop('Unnamed: 0', axis=1)
two_df = two_df.drop('Unnamed: 0', axis=1)
three_df = three_df.drop('Unnamed: 0', axis=1)
four_df = four_df.drop('Unnamed: 0', axis=1)
five_df = five_df.drop('Unnamed: 0', axis=1)
six_df = six_df.drop('Unnamed: 0', axis=1)
seven_df = seven_df.drop('Unnamed: 0', axis=1)
OG_df = OG_df.drop('Unnamed: 0', axis=1)

'''Calculate the relative percentage column and update it'''
one_df['Relative (%)'] = (one_df['Number']/(one_df['Number'].sum()+N))*100
two_df['Relative (%)'] = (two_df['Number']/(two_df['Number'].sum()+N))*100
three_df['Relative (%)'] = (three_df['Number']/(three_df['Number'].sum()+N))*100
four_df['Relative (%)'] = (four_df['Number']/(four_df['Number'].sum()+N))*100
five_df['Relative (%)'] = (five_df['Number']/(five_df['Number'].sum()+N))*100
six_df['Relative (%)'] = (six_df['Number']/(six_df['Number'].sum()+N))*100
seven_df['Relative (%)'] = (seven_df['Number']/(seven_df['Number'].sum()+N))*100
OG_df['Relative (%)'] = (OG_df['Number']/(OG_df['Number'].sum()+N))*100

'''Add a rank column - used for merge later'''
one_df['Rank'] = one_df['Relative (%)'].rank(ascending=False, method='first')
two_df['Rank'] = two_df['Relative (%)'].rank(ascending=False, method='first')
three_df['Rank'] = three_df['Relative (%)'].rank(ascending=False, method='first')
four_df['Rank'] = four_df['Relative (%)'].rank(ascending=False, method='first')
five_df['Rank'] = five_df['Relative (%)'].rank(ascending=False, method='first')
six_df['Rank'] = six_df['Relative (%)'].rank(ascending=False, method='first')
seven_df['Rank'] = seven_df['Relative (%)'].rank(ascending=False, method='first')
OG_df['Rank'] = OG_df['Relative (%)'].rank(ascending=False, method='first')

'''Find WT native AA sequence and one AA misreads of AETVESCLAKSH'''
one_df = OG_df[OG_df['AA'].str.contains('[A-Z]ETVESCLAKSH')==True]
two_df = OG_df[OG_df['AA'].str.contains('A[A-Z]TVESCLAKSH')==True]
three_df = OG_df[OG_df['AA'].str.contains('AE[A-Z]VESCLAKSH')==True]
four_df = OG_df[OG_df['AA'].str.contains('AET[A-Z]ESCLAKSH')==True]
five_df = OG_df[OG_df['AA'].str.contains('AETV[A-Z]SCLAKSH')==True]
six_df = OG_df[OG_df['AA'].str.contains('AETVE[A-Z]CLAKSH')==True]
seven_df = OG_df[OG_df['AA'].str.contains('AETVES[A-Z]LAKSH')==True]
eight_df = OG_df[OG_df['AA'].str.contains('AETVESC[A-Z]AKSH')==True]
nine_df = OG_df[OG_df['AA'].str.contains('AETVESCL[A-Z]KSH')==True]
ten_df = OG_df[OG_df['AA'].str.contains('AETVESCLA[A-Z]KH')==True]
elleven_df = OG_df[OG_df['AA'].str.contains('AETVESCLAK[A-Z]H')==True]
twelth_df = OG_df[OG_df['AA'].str.contains('AETVESCLAKS[A-Z]')==True]
```

```
'''Concat all df's'''
concatted_df = pd.concat([one_df, two_df, three_df, four_df, five_df, six_df, seven_df, eight_df, nine_df, ten_df, elleven_df, twelth_df], axis=0)

'''Drop duplicates rank columns in the concat df'''
dropduplicates_df = concatted_df['Rank'].drop_duplicates(keep='first')

'''Merge on Rank column into originl df'''
WT_df = pd.merge(dropduplicates_df, OG_df, on="Rank")

'''Print WT csv files'''
WT_df.to_csv('Removed_WT_t0.csv')
# WT_df.to_csv('Removed_WT_t150.csv')
# WT_df.to_csv('Removed_WT_t270.csv')

'''Percentages of WT + WT misreads of total removed reads'''
x = sum(WT_df.Number)
y = sum(OG_df.Number)
z = x/(y+N)*100
```
